# Wastewater-based assessment of episodic changes in psychoactive compounds and hormones during music festivals

**DOI:** 10.1007/s10661-026-15718-y

**Published:** 2026-07-24

**Authors:** Alexandra Tulipánová, Tomáš Mackuľak, Ivana Horáková, Zuzana Imreová, Alexandra Paulína Drdanová, Adam Bořík, Pavel Šauer, Hana Kocour Kroupová, Roman Grabic, Paula Bímová, Andrea Vojs Staňová

**Affiliations:** 1 Department of Environmental Engineering, Institute of Chemical and Environmental Engineering, Faculty of Chemical and Food Technology, Slovak University of Technology, Radlinského 9, Bratislava, 812 37 Slovakia; 2 Institute of Integral Safety, Faculty of Materials Science and Technology in Trnava, Slovak University of Technology in Bratislava, Jána Bottu, Trnava, 2781/25, 917 24 Slovakia; 3 Faculty of Fisheries and Protection of Waters, South Bohemian Research Center of Aquaculture and Biodiversity of Hydrocenoses, University of South Bohemia in České Budějovice, Zátiší 728/II, CZ-389 25 Vodňany, Czech Republic; 4 Department of Analytical Chemistry, Faculty of Natural Sciences, Comenius Universityin Bratislava, Ilkovičova 6, Bratislava, 842 15 Slovakia; 5 MicroPoll s.r.o., Vazovova 5, 812 43 Bratislava, Slovakia; 6 Research Centre 4D- National Research Centre for Defense and Dual Technologiesof the University of Ss. Cyril and Methodius in Trnava, Námestie J. Herdu 577/2, 917 01 Trnava, Slovakia

**Keywords:** Festivals, Progestagens, Illicit drugs, Psychoactive pharmaceuticals, Wastewater

## Abstract

This study investigated 11 psychoactive contaminants in wastewater influents at a municipal wastewater treatment plant in Slovakia between 2015 and 2018, based on 44 samples collected under regular conditions and an additional five samples collected during two music festivals in 2018. In addition, 17 progestagens were analyzed only in 2018 during festival days and their corresponding background days. Seasonal variation in normalized mass loads was generally minor, whereas interannual differences were more pronounced, particularly for psychoactive pharmaceuticals, which showed an overall decrease in 2018. Short-term but substantial increases in contaminant loads were observed during the festivals. During the multi-genre music festival, the normalized mass load of 3,4-methylenedioxymethamphetamine increased by more than 110-fold on the second festival day compared to the pre-festival average, while benzoylecgonine increased approximately 17-fold and dienogest by 1.3-fold. During the country/folk music festival, tramadol loads increased approximately twofold. Principal component analysis explained 70.75% of the total variance using the first two components and revealed a clear separation between festival and background samples. Overall, the results demonstrate that music festivals represent episodic pollution events capable of markedly altering wastewater influent composition over short time periods.

## Introduction

Prescription medications, illicit drugs, and pharmaceuticals that are frequently misused fall under a wide category of emerging pollutants (La Farre et al., 2008; Garcia et al., 2020). River ecosystems face significant risks of contamination from these pollutants when wastewater treatment plant (*WWTP*) effluents are not adequately treated (Jiang et al., 2024). Effluents containing these pollutants may subsequently impact aquatic life, leading to behavioral alterations, histopathological changes, biochemical imbalances, and disturbances in reproduction (Grabicová et al., 2018; Grabicová et al., 2022).

Music festivals are popular events that attract large crowds. Evidence from cross-sectional studies (De Almeida & Silva, 2005; Forsyth et al., 1997; Martin et al., 1993; Pedersen & Skrondal, 1999; Van Dyck et al., 2023), as well as biomarker screening in wastewater during festivals (Benaglia et al., 2020; Mackuľak et al., 2014, 2019; Senta et al., 2023), suggests a link between illicit drug use and the genre profile of festivals. Wastewater-based epidemiology (*WBE*) is an increasingly important monitoring approach that relies on the measurement of specific biomarkers in untreated wastewater to assess human activities at the population level. Over the past decade, WBE has become a well-established tool for monitoring the consumption of both legal and ilegal substances, offering high spatial and temporal resolution that traditional epidemiological methods often lack (Boogaerts et al., 2021; Lorenzo & Picó, 2019). While levels of psychoactive substances in wastewater remain stable during country, folk, and metal festivals, stimulants such as 3,4-methylenedioxymethamphetamine (MDMA) and cocaine tend to increase during dance, pop, and multi-genre events, indicating their higher consumption (Mackuľak et al., 2019). Similarly, Senta et al. (2023), in their study focusing on wastewater during a large electronic music festival in Split, reported a significant increase, with MDMA concentrations rising approximately 30-fold during the event. According to Festival Insights, over 70% of festival attendees in Europe were aged between 18 and 34 in 2016 (Festival Insights, 2017). Festivals featuring popular music styles—such as pop, indie, or electronic—like Lollapalooza, Glastonbury, or Sziget, are especially appealing to young women, many of whom are in their reproductive years (Festivalpro, 2022; Viberate Analytics, 2022). This group is also a key user of hormonal therapies, particularly progestagens such as natural progesterone or synthetic progestins. These compounds are commonly prescribed in contraceptives, hormone replacement treatments for menopause, fertility therapies, and other hormone-related conditions (Schindler, 2009; Sitruk-Ware, 2008). Both progestagens and their metabolites have been found in wastewater and surface waters in several countries (Golovko et al., 2018; Rocha & Rocha, 2022; Šauer et al., 2018), where they were suspected of posing a threat to the aquatic ecosystem. These, often highly biologically active, substances can interfere with reproduction, behavior, and development in various aquatic species (Jenila et al., 2023; Orlando & Ellestad, 2014).

Therefore, the objectives of this work were:

- to analyze wastewater samples collected at the *WWTP* during the years 2015, 2016, and 2018 for the presence of illicit drugs, their metabolites, and psychoactive pharmaceuticals, and to statistically evaluate the mass loads in terms of seasonal and interannual variability;
- to analyze wastewater samples collected at the same *WWTP* during two different music festivals by screening for the presence of the aforementioned contaminants as well as additional hormonal contaminants (progestagens);
- to assess whether these events had a significant impact on the daily mass loads of the contaminants, including statistical evaluation using principal component analysis (*PCA*);
- to discuss the ability of the *WWTP* to remove these contaminants;

## Materials and methods

### Reagents and materials

Ultrapure water, methanol, and acetonitrile of LC-MS grade were obtained from Merck (Darmstadt, Germany). Formic acid for mobile phase acidification and regenerated cellulose injection filters were supplied by Labicon (Olomouc, Czech Republic). Glass fiber prefilters and C18 SPE disks were provided by Horizon Technology (U.S.). Progestagens, illicit drugs, and psychoactive pharmaceuticals, selected as target analytes from Sigma-Aldrich (St. Louis, MO, U.S.) and AK Scientific (Union City, U.S.). The internal standards (0.1 or 1 mg/L in methanol or acetonitrile) for progestagen analysis were obtained from Toronto Research Chemicals (ON, Canada); for illicit drugs and psychoactive pharmaceuticals, they were obtained from Cerilliant (Round Rock, Texas, U.S.) and Chiron (Trondheim, Norway). Information about target analytes and internal isotopically labeled standards is available in Tables SI1 and SI2.

### Description of sampling, investigated events, and WWTP characterization

Between 2015 and 2018, a total of 44 influent wastewater samples were collected at a *WWTP* in western Slovakia during regular operation (Table SI3). The year 2017 was excluded from the evaluation due to the absence of samples from the summer period. The *WWTP* has a design capacity of 70,000 population equivalents (*PE*). However, during the study period the actual load of the plant corresponded to approximately 30,000 *PE*, based on the number of connected inhabitants and the operational load of the *WWTP* estimated from biochemical oxygen demand values (Mackuľak et al., 2019). The treatment process is based on conventional activated sludge technology, with no tertiary or quaternary treatment applied. The average wastewater conveyance time in the sewer system was approximately 6 h. Sampling was performed using automatic devices set to collect time-proportional aliquots every 15 min over a 24-h period, with sampling starting at 07:00 h. All samples were subsequently frozen at −20 °C and stored in the dark until analysis (Aboulfadl et al., 2010; Ort et al., 2014).

Additionally, five wastewater samples were collected during two music festivals held in 2018 (Table SI3, festival sampling days are shown in bold): a multi-genre music festival (*MGF*) with approximately 25,000 attendees, organized on 10–11 August, and a country/folk music festival (*CFF*) with approximately 10,000 attendees on 30 August–1 September. For both festivals, wastewater originated exclusively from mobile chemical toilets. Wastewater accumulated in a holding tank throughout the day and was discharged directly into the *WWTP* influent the following day. Because formaldehyde, a biological process inhibitor, is commonly used in chemical toilets, the tank contents were gradually diluted by the subsequent daily inflow (Mackuľak et al., 2019). Consequently, due to the delayed input of wastewater from mobile chemical toilets, the initial days of both festivals were considered representative of background conditions. Accordingly, in this study, 31 August was considered the first day of the *CFF*, and 11 August was considered the first day of the *MGF*.

### Instrumental analysis

Quantitative analysis of psychoactive substances was performed using in-line solid-phase extraction and liquid chromatography–tandem mass spectrometry. 10 mL of filtered wastewater (0.20 μm regenerated cellulose syringe filter) was spiked with a 1 μg/mL standard mixture and stored at −20 °C. The use of isotope dilution along with internal standards helped to limit the impact of matrix effects. A 1 mL aliquot was applied to a Hypersil Gold aQ SPE column (20 × 2.1 mm, 12 μm) and subsequently eluted with an acetonitrile–water mixture containing 0.1% formic acid (see Table SI4). The eluate was subsequently delivered to a Hypersil Gold aQ analytical column (50 × 2.1 mm, 5 μm). Compound detection was carried out using a TSQ Quantiva triple quadrupole mass spectrometer supplied by Thermo Fisher Scientific (San Jose, CA, USA). Further details are provided in Table SI5. Method details, including validation, are previously published in Fedorova et al. (2013) and Fedorova et al. (2014). A summary of validation parameters of applied instrumental methods for the analysis of illicit drugs and psychoactive pharmaceuticals (linearity, *LOD*, *LOQ*, recovery, and matrix effects), based on the publications cited above, is provided in Table SI6.

Quantitative analysis of progestagens was performed using liquid chromatography tandem atmospheric pressure chemical ionization/atmospheric pressure photoionization with hybrid quadrupole/orbital trap mass spectrometry operated in high resolution product scan mode. Briefly, 1 L wastewater samples were filtered through 5 μm, and 1 μm glass fiber and passed through an Atlantic C18 sorbent. After air-drying, analytes were eluted with acetonitrile, evaporated under nitrogen (Termovap TV10+, ECOM, Czech Republic), and re-dissolved in 100 μL acetonitrile. Extracts were analyzed using a Hypersil Gold column (50 mm × 2.1 mm, 3 μm, Thermo Fisher Scientific) with methanol–water gradient elution (more information is given in Table SI7 and SI8). Details on method validation and performance are in Golovko et al. (2018) and Šauer et al. (2024). A summary of validation parameters of applied instrumental methods for the analysis of progestagens (linearity, *LOD*, *LOQ*, recovery, and matrix effects), based on the publications cited above, is provided in Table SI9.

### Data normalization and statistical analysis

Daily contaminant mass loads (*M*_*i,d*_) in mg/day were calculated from measured concentrations (*C*_*i*_) in ng/day and corresponding daily inflow volumes (*Q*_*d*_) in m^3^/day according to Eq. (1). Daily contaminant mass loads were normalized (*M*_*i,d,norm*_) to 1,000 *PE*, assuming a population of 30,000 *PE* according to Eq. (2), with festival participants included in the total population during festival days.

1 $${M}_{\mathrm{i},\mathrm{d}}=\frac{{C}_{i}\times {Q}_{d}}{\mathrm{1,000}}$$

2 $${M}_{\mathrm{i},\mathrm{d},\mathrm{n}\mathrm{o}\mathrm{r}\mathrm{m}}={M}_{\mathrm{i},\mathrm{d}}\times \frac{\mathrm{1,000}}{\mathrm{30,000}}$$

Seasonal (spring vs. summer) and interannual differences in mass loads of illicit drugs and psychoactive pharmaceuticals were assessed using the Mann–Whitney *U* test. Results are reported as *p*-values, with the significance level (α) set at 0.05. In cases where measured concentrations were below *LOQ*, values were substituted with LOQ/2. The Mann–Whitney *U* tests were performed using GraphPad Prism software (version 10.4.2; GraphPad Software, San Diego, CA, USA). *PCA* was applied to explore multivariate patterns among wastewater samples collected before, during, and after the music festivals and was conducted using OriginPro 2025 software (OriginLab Corporation, Northampton, MA, USA).

## Results and discussion

### Seasonal and interannual variations in mass load

A total of eleven contaminants were monitored in influent at the selected *WWTP* to assess their occurrence, mass loads, and normalized mass loads. These included illicit drugs such as methamphetamine (MET), amphetamine (AMP), cocaine (COC), benzoylecgonine (BEN), MDMA, and 11-nor-9-carboxy-tetrahydrocannabinol (THC-COOH), as well as psychoactive substances including codeine (COD), tramadol (TRA), venlafaxine (VEN), oxazepam (OXA), and citalopram (CIT). Table SI10 in the supplementary information summarizes the detection frequency in the wastewater samples collected during the spring and summer seasons in 2015, 2016, and 2018. AMP, MET, COD, TRA, VEN, OXA, and CIT were detected in all analyzed samples, indicating their consistent presence. In contrast, MDMA, COC, and its metabolite BEN were not consistently detected across all sampling periods, with the lowest detection frequency (0%, 14%, 29%) observed in spring 2015. Quantitatively, the highest normalized daily mass loads were recorded for MET among illicit drugs (Table 1), and for TRA among psychoactive pharmaceuticals (Table 2).

**Table 1:** Normalized daily mass loads (mg/day.1,000 *PE*) of illicit drugs and their metabolites by year and season

Year	Season	Median [Q1-Q3]					
		**AMP**	**MET**	**BEN**	**COC**	**MDMA**	**THC-COOH**
2015	Spring	23 [16–31]	140 [97–180]	9.5 [8.8–10]	5.8 [5.8–5.8]	-	22 [19–28]
	Summer	13 [8.6–20]	220 [100–390]	5.7 [4.4–7.5]	9.4 [7.6–11]	48 [27–68]	31 [25–34]
2016	Spring	29 [27–34]	290 [270–350]	14 [12–28]	8 [5.5–15]	7 [6.3–15]	22 [21–32]
	Summer	12 [12–13]	160 [150–280]	15 [13–17]	5.5 [4.7–6.5]	4.8 [4.7–6.8]	10 [7.0–13]
2018	Spring	24 [17–31]	93 [52–110]	10 [9.5–29]	0.81 [0.64–0.91]	2.2 [1.7–3.0]	17 [12–23]
	Summer	33 [28–42]	190 [170–220]	22 [16–48]	8.8 [7.0–16]	6.3 [4.6–20]	28 [22–38]

**Table 2:** Normalized daily mass loads (mg/day.1,000 *PE*) of psychoactive pharmaceuticals by year and season

Year	Season	Median [Q1-Q3]				
		**COD**	**TRA**	**VEN**	**OXA**	**CIT**
2015	Spring	39 [31–49]	420 [370–490]	110 [93–130]	25 [22–38]	48 [43–49]
	Summer	25 [19–31]	140 [100–160]	68 [35–100]	20 [15–26]	37 [32–49]
2016	Spring	15 [15–20]	390 [320–430]	43 [38–62]	19 [15–20]	41 [39–48]
	Summer	29 [20–37]	300 [270–330]	99 [74–130]	44 [29–54]	44 [29–49]
2018	Spring	21 [11–25]	100 [56–140]	21 [16–39]	7.7 [5.4–9.6]	14 [10–19]
	Summer	24 [23–38]	180 [170–270]	44 [42–50]	12 [11–13]	20 [19–28]

The elevated levels of MET in influents compared to other illicit drugs observed in this study likely reflect its high prevalence, particularly in the western region of Slovakia (Mackuľak et al., 2014). TRA exhibited the highest normalized daily mass loads among the monitored pharmaceuticals across all investigated years (Table 2). This may be partly explained by the combination of its high nationally dispensed amounts, ranging from 2680 to 3160 kg/year during the investigated period (Table 3), and its relatively high unchanged urinary excretion fraction of approximately 20–30% of the administered dose (Bamfo et al., 2021; Grond & Sablotzki, 2004). In contrast, the proportions excreted unchanged in urine are lower for the other monitored compounds, amounting to approximately 12% for CIT, less than 5% for VEN, and less than 1% for both COD and OXA (Bamfo et al., 2021). Despite its low unchanged urinary excretion fraction, VEN was dispensed in relatively high amounts, ranging from 917 to 978 kg/year, which may partly contribute to its comparatively elevated normalized daily mass loads.

**Table 3:** Quantities of dispensed psychoactive pharmaceuticals belonging to the Anatomical Therapeutic Chemical classification for clinical use in the Slovak Republic in 2015–2018 given out by the National Health Information Center website (NCZISK, 2024)

Main group	Therapeutic subgroup	Compound	Dispensed amount* (kg/year)		
			**2015**	**2016**	**2018**
Nervous system	Psychoanaleptics	CIT	242	238	236
		VEN	917	972	978
	Psycholeptics	OXA	56.0	55.9	56.7
	Analgesics	COD	15.3	15.2	12.9
		TRA	3,160	3,070	2,680
Respiratory system	Expectorants, Mucolytics and Antitussives	COD	31.2	27.5	32.2

*These national-level data are presented for contextual comparison only and are not specific to the catchment area of the investigated WWTP

**Fig. 1:**
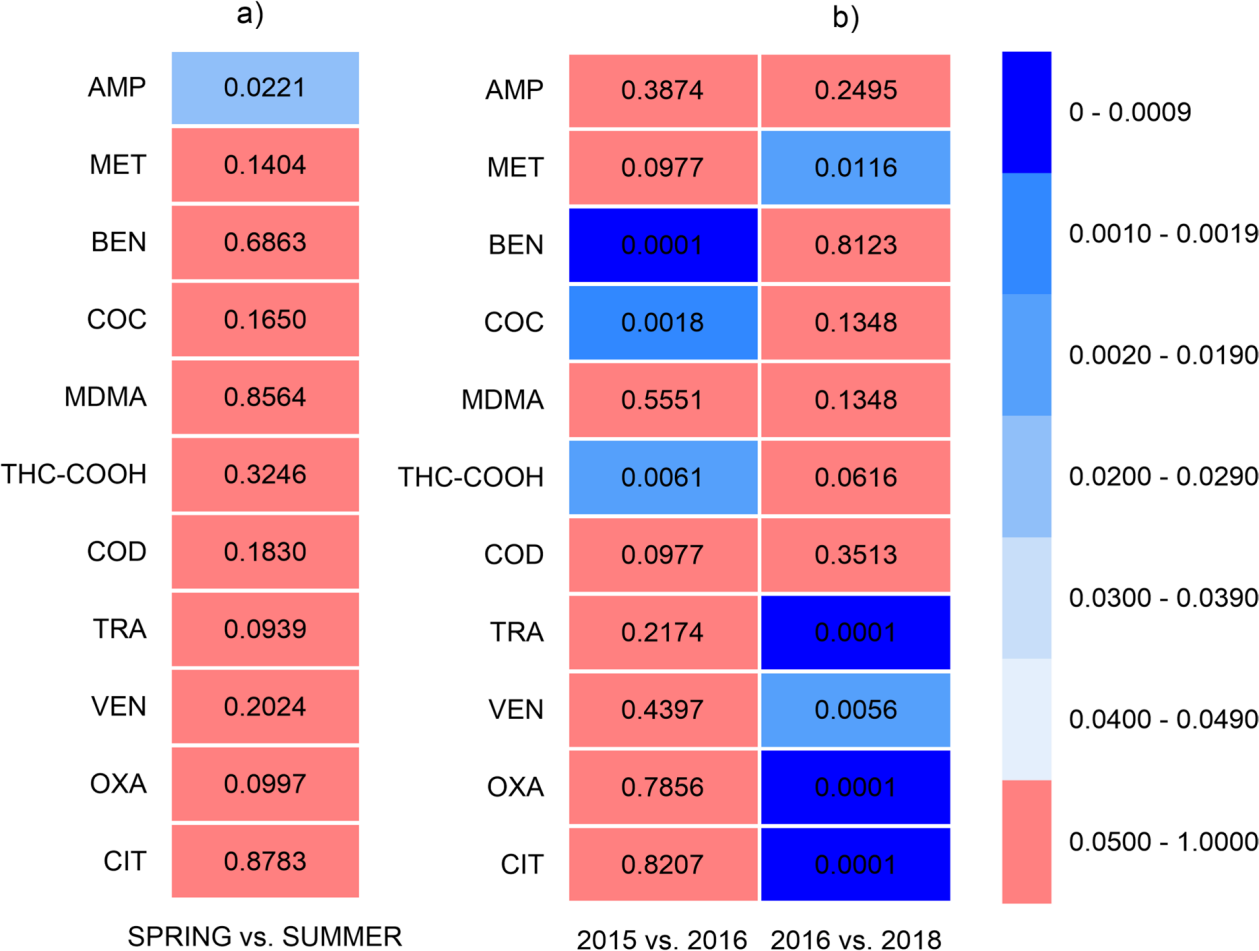
Heat map of *p*-values from the Mann–Whitney *U* test comparing *WWTP* mass loads of the analyzed compounds: **a** seasonally, **b** interannually

**Fig. 2:**
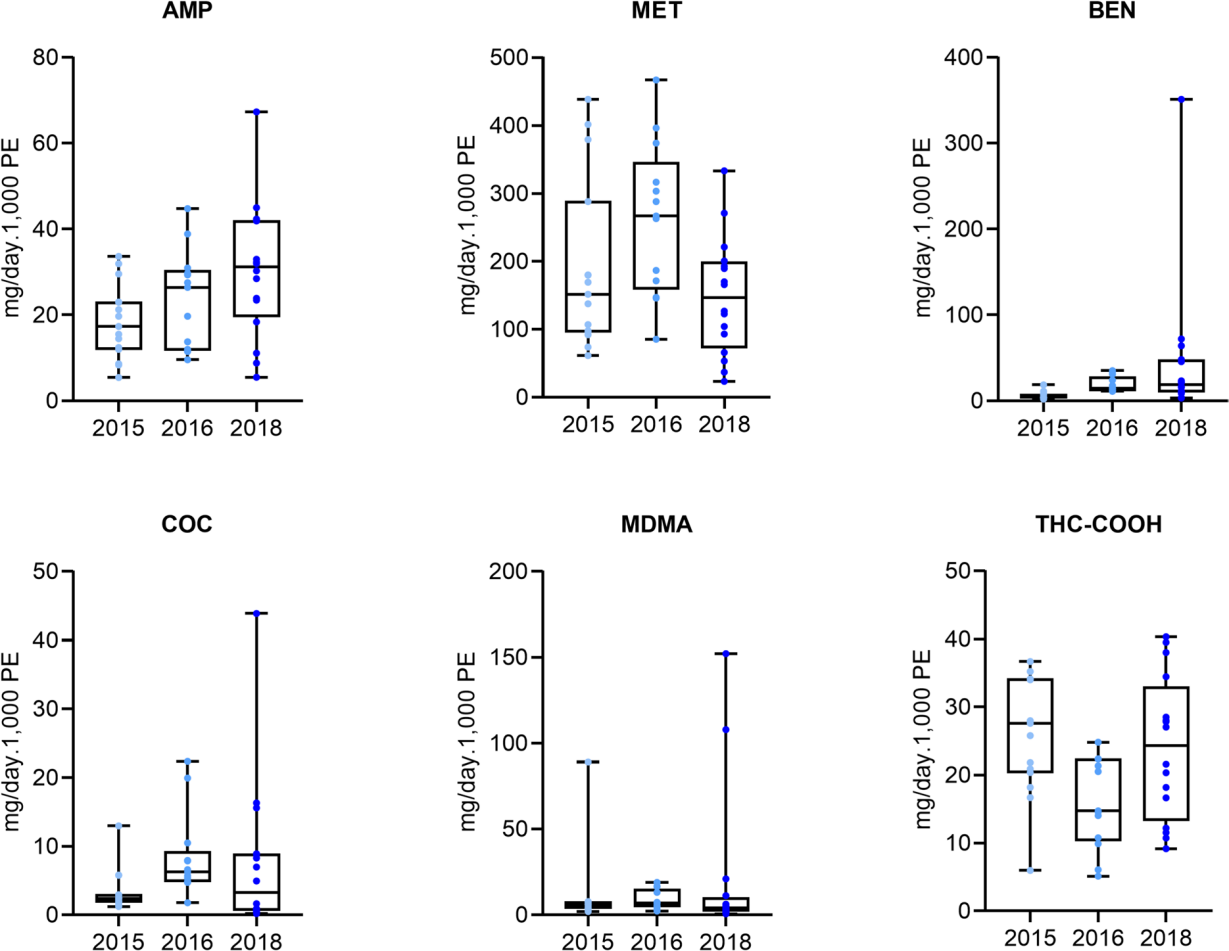
Interannual variation in normalized contaminant mass loads from the analyzed group of illicit drugs

**Fig. 3:**
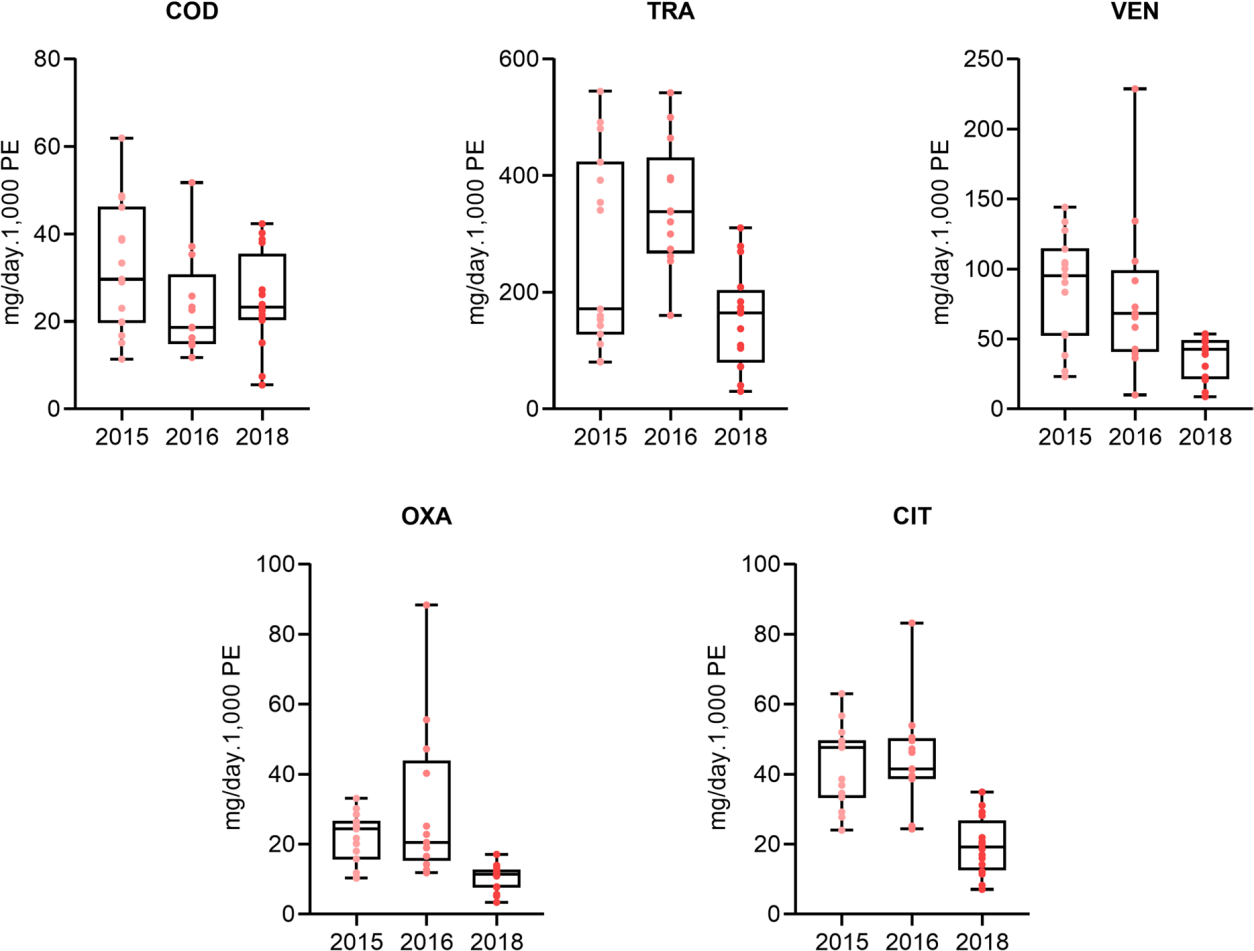
Interannual variation in normalized contaminant mass loads from the analyzed group of psychoactive pharmaceuticals

Seasonal (spring vs. summer) and interannual differences in normalized contaminant mass loads were evaluated using the Mann–Whitney *U* test, which was applied due to the non-normal distribution of the data. Results are presented as *p*-values in Fig. 1. Of all the monitored drug residues, a statistically significant seasonal difference (*p* < 0.05) was found only for AMP, with higher mass loads recorded in the spring (Fig. 1a). As AMP is poorly biodegradable and not subject to significant degradation in the sewer system (Van Nuijs et al., 2012), elevated summer temperatures are unlikely to explain the observed seasonal decrease. The higher mass loads in spring are therefore more likely linked to increased AMP consumption during this period. However, AMP can also be formed as a metabolite of other substances included in this study, particularly MET (Thai et al., 2014), making it difficult to clearly determine whether the AMP originates from direct use or metabolic transformation. Addressing this uncertainty would require a more refined approach, such as enantiomeric analysis, as demonstrated in the study by Sharfudeen et al. (2023). Although a statistically significant seasonal difference in AMP mass load was observed, no significant changes were found interannually (Fig. 1b). This suggests a relatively stable pattern of AMP use within the population during the study period, without major annual fluctuations. Notable interannual differences were observed for COC (Fig. 1b). This difference is driven by an increase in mass loads in 2016, as illustrated in Fig. 2, which shows the interannual variation in normalized mass loads of illicit drugs and their metabolites. This pattern was mirrored by BEN, its primary metabolite. A statistically significant interannual difference was also observed for THC-COOH, which showed a decrease between 2015 and 2016 (Fig. 2). MET also showed significant interannual differences (Fig. 1b), characterized by higher mass loads in 2018 compared to 2016 (Figs. 2). In contrast to illicit drugs, psychoactive pharmaceuticals exhibited a more consistent interannual pattern. The most prominent changes between 2016 and 2018 were observed for this group of compounds (Fig. 1b), with a clear decrease in normalized mass loads observed in 2018 for TRA, VEN, OXA, and CIT (Fig. 3). Figure 3 illustrates the interannual variation in normalized mass loads of psychoactive pharmaceuticals, with lower median values observed in 2018 compared to previous years. For TRA, this decrease may be related to a lower dispensed quantity, whereas prescription amounts for VEN, OXA, and CIT remained relatively steady over the study period (Table 3).

### Short-term variations in mass load during music festivals

Among illicit drugs and their metabolites, six of the eight target analytes were found above the *LOQ*. All wastewater samples contained psychoactive pharmaceuticals above the *LOQ*. In terms of illicit drugs and their metabolites, there was a significant increase in the daily mass load of MDMA, THC-COOH, COC, and BEN during the *MGF* (Table 4). The MDMA load increased from a pre-festival average of 118 mg/day to 4,600 mg/day on the first day and up to 48,000 mg/day during the second day of the festival. The COC load showed a similar pattern, rising from 180 mg/day to 1,200 mg/day and 3,400 mg/day. However, COC consumption is better monitored based on the detection of its major metabolite BEN, as only 1–9% is excreted unchanged (Huizer et al., 2021). The daily BEN mass load increased from an average of 665 before the festival to 4,600 mg/day and peaked at 21,000 mg/day during the second festival day. Similarly, cannabis use was monitored based on its dominant metabolite THC-COOH (Bijlsma et al., 2024), with an increase in daily load from an average of 710 to 2,600 mg/day during the second festival day. The increase in MDMA, COC, and BEN mass load during *MGF* is consistent with previous research showing elevated MDMA and BEN levels during music festivals, particularly those with electronic, pop, or multi-genre line-ups (Benaglia et al., 2020; Mackuľak et al., 2019; Senta et al., 2023). Changes in daily mass load of psychoactive pharmaceuticals during *MGF* were minimal (Table 4).

**Table 4:** Daily mass loads of contaminants before, during, and after the *MGF*. Festival days are shown in bold, and normalized mass load values per 1000 *PE* are given in brackets

Contaminant	Mass load (mg/day)					
	09/08	10/08	**11/08**	**12/08**	13/08	14/08
DIE	25 (0.82)	20 (0.67)	**28 (0.50)**	**55 (1.0)**	25 (0.82)	8.9 (0.30)
P4	330 (11)	290 (9.6)	**700 (13)**	**580 (10)**	410 (14)	370 (12)
MGA	28 (0.93)	25 (0.84)	**55 (1.0)**	**19 (0.35)**	35 (1.2)	63 (2.1)
AMP	990 (33)	960 (32)	**1,700 (32)**	**1,600 (28)**	2,000 (67)	1,300 (42)
MET	5,700 (190)	5,000 (170)	**9,300 (170)**	**8,200 (150)**	5,800 (190)	6,000 (200)
BEN	630 (21)	700 (23)	**4,600 (84)**	**21,000 (380)**	11,000 (350)	1,400 (48)
COC	150 (5.0)	210 (7.0)	**1,200 (22)**	**3,400 (62)**	1,300 (44)	470 (16)
MDMA	140 (4.7)	96 (3.2)	**4,600 (84)**	**48,000 (870)**	4,600 (150)	3,200 (110)
THC-COOH	610 (20)	810 (27)	**990 (18)**	**2,600 (47)**	1,100 (38)	650 (22)
COD	780 (26)	630 (21)	**900 (16)**	**890 (16)**	710 (24)	680 (23)
TRA	6,300 (210)	5,200 (170)	**7,800 (140)**	**4,700 (85)**	4,100 (140)	4,900 (160)
VEN	1,500 (50)	1,300 (44)	**2,000 (37)**	**1,700 (32)**	1,300 (44)	1,300 (42)
OXA	370 (12)	340 (11)	**540 (10)**	**470 (8)**	360 (12)	380 (13)
CIT	610 (20)	590 (20)	**800 (15)**	**550 (10)**	510 (17)	560 (19)

In contrast to the *MGF*, the changes in daily mass load of illicit drugs observed during the *CFF* were generally more moderate (Table 5). Nevertheless, on the second day of the event, notable increases were recorded for MET (from a mean value of 7,350 to 15,000 mg/day), BEN (from a mean value of 480 to 3,500 mg/day), COC (from a mean value of 265 to 1,300 mg/day), MDMA (from a mean value of 265 to 900 mg/day), and THC-COOH (from a mean value of 1,100 to 3,200 mg/day). In the case of selected psychoactive pharmaceuticals, an increase in mass load corresponding to the second festival day was evident. Specifically, TRA (from a mean value of 8,700 to 19,000 mg/day), VEN (from a mean value of 1,450 to 3,500 mg/day), OXA (from a mean value of 365 to 720 mg/day), and CIT (from a mean value of 885 to 1,800 mg/day).

**Table 5:** Daily mass loads of contaminants before, during, and after the *CFF*. Festival days are shown in bold, and normalized mass load values per 1000 *PE* are given in brackets

Contaminant	Mass load (mg/day)						
	29/08	30/08	**31/08**	**01/09**	**02/09**	03/09	04/09
DIE	17 (0.56)	6.8 (0.23)	**19 (0.47)**	**31 (0.76)**	**16 (0.39)**	18 (0.60)	6.5 (0.22)
P4	230 (7.6)	230 (7.8)	**210 (5.3)**	**580 (15)**	**670 (17)**	740 (25)	290 (9.7)
MGA	28 (0.93)	25 (0.83)	**23 (0.58)**	**48 (1.2)**	**45 (1.1)**	26 (0.85)	13 (0.43)
AMP	1,300 (42)	850 (28)	**1,100 (27)**	**2,100 (52)**	**4,500 (110)**	1,300 (45)	550 (18)
MET	8,100 (270)	6,600 (220)	**7,200 (180)**	**15,000 (370)**	**7,900 (200)**	10,000 (330)	5,100 (170)
BEN	490 (16)	470 (16)	**980 (24)**	**3,500 (88)**	**1,400 (34)**	2,200 (72)	650 (22)
COC	260 (8.8)	270 (8.9)	**600 (15)**	**1,300 (32)**	**480 (12)**	490 (16)	250 (8.3)
MDMA	340 (11)	190 (6.3)	**220 (5.6)**	**900 (22)**	**550 (14)**	630 (21)	140 (4.6)
THC-COOH	1,200 (40)	1,000 (34)	**1,500 (38)**	**3,200 (80)**	**1,400 (35)**	1,200 (40)	840 (28)
COD	1,300 (42)	1,200 (40)	**1,100 (27)**	**1,900 (48)**	**960 (24)**	1,200 (39)	720 (24)
TRA	9,300 (280)	8,100 (270)	**8,800 (220)**	**19,000 (480)**	**7,600 (190)**	8,400 (280)	5,500 (180)
VEN	1,600 (54)	1,300 (43)	**1,500 (38)**	**3,500 (88)**	**1,500 (38)**	1,600 (53)	1,200 (39)
OXA	400 (13)	330 (11)	**370 (9.3)**	**720 (18)**	**400 (10)**	510 (17)	340 (11)
CIT	930 (31)	840 (28)	**890 (22)**	**1,800 (44)**	**700 (18)**	1,000 (35)	620 (21)

Influent samples from both festivals and the associated background contained analytes above *LOQ* for dienogest (DIE), progesterone (P4), and megestrol acetate (MGA). Concentrations of the other progestagens did not exceed the *LOQ* in any of the analyzed samples. Increased P4 load was observed during both festivals (Tables 4 and 5), especially on the first day of *MGF* (700 mg/day) and the last day of *CFF* (670 mg/day). The average daily loads of P4 during the pre-festival background period were 310 and 230 mg/day, respectively. DIE and MGA were also detected in wastewater samples. While no significant change in MGA loads was observed compared to background days, DIE loads increased on the second day of the *MGF* (from an average of 23 to 55 mg/day). The synthetic progestagen DIE is used exclusively in human medicine, primarily as part of hormonal contraception prescribed to women of reproductive age (Ruan et al., 2012). DIE is predominantly excreted as a metabolite, mainly 11β-hydroxydienogest, which retains only approximately 2% of the progestagenic activity of the parent compound (Pflug et al., 2017). These facts may explain the lower mass load of DIE compared to P4.

**Fig. 4:**
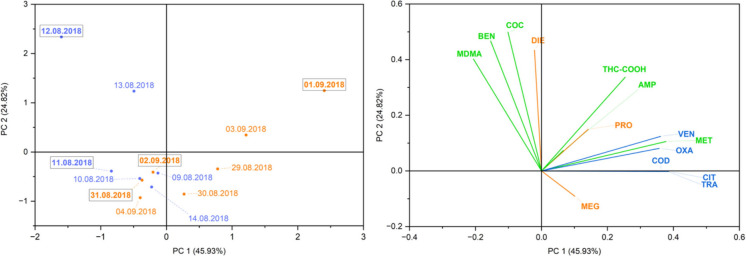
*PCA* of normalized daily mass loads of contaminants before, during, and after music festivals: **a** score plot (*MGF* is shown in blue, *CFF* in orange, with festival days in bold), **b** loading plot (illicit drugs and metabolites are shown in green, psychoactive pharmaceuticals in blue, and progestagens in orange)

*PCA* was used to visualize shifts in the chemical profiles of wastewater and to highlight changes in the mass loads of selected substances during both types of music festivals. The input data consisted of Z-score standardized daily mass loads (mg/day·1,000 *PE*) of psychoactive substances and progestagens. The *PCA* score plot (Fig. 4a) revealed a clear separation between festival days and pre-/post-festival background days. The second day of the *MGF* clearly appears as an outlier in the score plot (Fig. 4a), showing a strong positive association with principal component 2 (*PC2*). *PC2* explains 24.82% of the total variance and is primarily driven by MDMA, COC, BEN, and DIE, as shown in the loading plot (Fig. 4b). Similarly, the second day of the *CFF* deviated from the cluster of background samples, showing a positive association with principal component 1 (*PC1)* (Fig. 4a). *PC1* explains 45.93% of the total variance and is primarily driven by CIT, TRA, MET, and VEN (Fig. 4b). Together, *PC1* and *PC2* explain 70.75% of the total variance, indicating that the separation of festival days from background samples is supported by a substantial proportion of the overall data variability. The distinct positioning of the second days of both festivals suggests pronounced, compound-specific shifts in wastewater composition during peak festival activity. The post-festival days (13/08 for *MGF* and 03/09 for *CFF*) also showed noticeable deviation from the cluster of background days, which may reflect residual effects of increased pollutant loading. In the case of the *CFF*, this shift may have been further influenced by higher daily inflow volumes associated with weather conditions (storms and heavy rainfall) (Table SI3). According to information provided by event organizers, the *MGF* attracts a younger audience (average age below 28 years), whereas the *CFF* tends to attract an older demographic (average age around 35 years). Such demographic differences may partly contribute to the observed variation in the occurrence and intensity of selected substances between the two events. For example, elevated DIE loads may be associated with a higher proportion of women of reproductive age during the *MGF*. Overall, these demographically driven and episodic increases in pharmaceutical and illicit drug loads can measurably alter *WWTP* influent composition and may have implications for the ecological status of receiving waters.

### Removal in WWTPs

Based on the previously discussed results, music festivals may cause short-term but significant increases in the loads of psychoactive and hormonal micropollutants. Reported removal efficiencies by activated sludge vary widely: 46–100% (BEN), 72–100% (COC), 59–100% (AMP), 63–100% (MET), 19–36% (MDMA) and 31–98% (THC-COOH) (Bones et al., 2007; Boleda et al., 2009; Kasprzyk-Hordern et al., 2009; Loganathan et al., 2009; Postigo et al., 2010; Bijlsma et al., 2012; Baker & Kasprzyk-Hordern, 2013; Chiavola et al., 2017). Removal depends on substance-specific properties and operational conditions, with sorption to activated sludge as the dominant pathway (Mackuľak et al., 2016; Yadav et al., 2017). Psychoactive pharmaceuticals are poorly removed without advanced treatment: 16–74% (COD), 10–40% (TRA), <50% (VEN), and <75% (CIT) (Ding & Zhang, 2024; Irikura et al., 2025; Restrepo-Vieira et al., 2024; Simpson et al., 2024), partly due to transformation products (Wang et al., 2023), with sorption again prevailing (Santana-Viera et al., 2023). Increased TRA, VEN, and CIT loads during the *CFF* indicate likely discharge into receiving surface waters. Progestagen removal is variable; although rapid degradation is observed in lab conditions, DIE is more persistent (Weizel et al., 2021). Field data from *WWTP*s in the Czech and Slovak Republics report activated sludge removal efficiencies of 77–100% (P4), 73–100% (MGA), and 76–100% (DIE) (Šauer et al., 2018).

## Conclusions

The long-term monitoring results showed that seasonal variation in contaminant mass loads was generally not statistically significant, except for amphetamine, which exhibited higher loads in spring. In contrast, interannual differences were more pronounced, particularly for psychoactive pharmaceuticals, where all monitored substances except codeine showed a decrease in 2018. In the case of tramadol, this decline may be associated with a lower dispensed quantity in that year. Music festivals were identified as episodic events causing short-term but significant increases in the loads of selected illicit drugs, psychoactive pharmaceuticals, and progestagens in wastewater influent. The magnitude and chemical profile of these increases differed between events. Based on the presented results, these differences appear to be influenced by the music genre and the demographic characteristics of festival attendees. These findings demonstrate that mass events can substantially alter *WWTP* influent composition over short time periods.

## Limitations of this study and future perspectives

This study has several limitations that should be considered when interpreting the results. Sampling was conducted during two mass events, which enables their direct comparison and the identification of differences between festival types. However, the scope of the study is limited to these specific events; therefore, caution should be exercised when extrapolating the findings to a broader context.

Another potential limitation is the origin of the analyzed wastewater from chemical toilet systems, where biocidal additives such as formaldehyde may be present. Formaldehyde is widely used in these systems (Crous & Haarhoff, 2011) and is a chemically reactive compound capable of interacting with nucleophilic functional groups, particularly primary and secondary amines, leading to the formation of Schiff bases. In the presence of formic acid, additional transformation reactions may occur, resulting in the formation of modified (e.g., N-methylated) derivatives (Takayasu, 2013; Zhou et al., 2025). From this perspective, analytes containing primary or secondary amine functional groups (e.g., AMP, MET, MDMA, and structurally related compounds) may be particularly susceptible to such reactions, whereas compounds containing tertiary amines or lacking free amine groups are likely less prone to direct chemical interactions with formaldehyde. Experimental studies in formalin-based systems indicate that under high formaldehyde concentrations (e.g., ~15% formalin, pH 4.5–5) and extended exposure times (days to weeks), decreases in amphetamine concentrations may occur, primarily due to their transformation into modified derivatives. At the same time, these compounds have been shown to undergo changes in formalin matrices while remaining detectable even after prolonged exposure (Takayasu, 2013; Uekusa et al., 2015). Such conditions, however, represent an extreme scenario compared to real conditions in chemical toilet systems. In chemical toilet products, formaldehyde concentrations have been reported in the range of 34.1–1327 mg/L, which, after typical dilution (1:20), correspond to approximately 1.7–66 mg/L, representing maximum theoretical concentrations in the holding tank. Under real conditions, these concentrations are expected to decrease further due to continuous input of urine and fecal matter, as well as biological activity and gradual degradation of formaldehyde (Crous & Haarhoff, 2011). In addition, the complex wastewater matrix, rich in organic and nitrogen-containing compounds, provides numerous competing reactive substrates, which may further limit the interaction of formaldehyde with trace-level biomarkers. Overall, while some degree of transformation of the analyzed compounds—particularly those containing amine groups—cannot be completely excluded, the substantially lower formaldehyde concentrations under real conditions, its rapid degradation, and the strong influence of matrix effects suggest that its impact on the measured concentrations is likely limited.

This study focused exclusively on influent wastewater and did not include effluent analysis. As a result, the removal efficiency of the investigated compounds could not be directly assessed, and available information on their elimination is based primarily on literature data. Given that the removal of micropollutants in activated sludge systems is known to be highly variable and dependent on operational conditions, it is not possible to reliably determine the extent to which episodic load increases are attenuated by the treatment process. Consequently, it cannot be excluded that such short-term peaks may lead to increased discharge of these substances into receiving waters.

The observed episodic increases in contaminant loads highlight the importance of targeted monitoring during mass events. Future studies should therefore include simultaneous monitoring of *WWTP* effluent during such events to evaluate how short-term load increases are handled by activated sludge treatment systems and whether they lead to increased discharge to receiving waters. In addition, systematic long-term monitoring of hormonally active compounds is needed to assess their seasonal and interannual variability and to better understand their behavior in wastewater systems.

## Data Availability

No datasets were generated or analyzed during the current study.

## Abbreviations

AMP: Amphetamine
BEN: Benzoylecgonine
*CFF*: Country/folk music festival
*C*_*i, d*_: Measured concentration of contaminant in the influent wastewater
CIT: Citalopram
COC: Cocaine
COD: Codeine
DIE: Dienogest
MDMA: 3,4-Methylenedioxymethamphetamine
MET: Methamphetamine
MGA: Megestrol acetate
*MGF*: Multi-genre music festival
*M*_*i,d*_: Daily mass loads
*M*_*i,d,norm*_: Normalized daily mass loads
OXA: Oxazepam
P4: Progesterone
*PCA*: Principal component analysis
*PC1*: Principal component 1
*PC2*: Principal component 2
*PE*: Population equivalents
*Q*_*d*_: Daily wastewater inflow volume
THC-COOH: 11-Nor-9-carboxy-tetrahydrocannabinol
TRA: Tramadol
VEN: Venlafaxine
*WBE*: Wastewater-based epidemiology
*WWTP*: Wastewater treatment plant
